# Supplementary material for: HIVprotI: an integrated web based platform for prediction and design of HIV proteins inhibitors
Source: J Cheminform. 2018 Mar 9;10:12. doi: 10.1186/s13321-018-0266-y (PMC5845081; doi:10.1186/s13321-018-0266-y)
Supplement: Supplementary file 2 — Additional file 2. Source code of HIVProtI web server. [file 13321_2018_266_MOESM2_ESM.zip › HIVprotI_Source-code/hivprotI/jsme/JSME_dnd_demo.html]

# JSME drag and drop

If drag and drop is supported by your browser, an icon will be displayed at the bottom right of the editor.


### Testing the DnD capabilities of JSME:

1. Draw a structure on the top editor
2. Drag it out and drop it on the surface of the bottom editor
3. Drag it out and drop it on your computer local file system to save it (does not work in Windows)
4. Drag it out and drop it on the window of another program, e.g. text editor
5. Drag a MOL or SDF file from your computer local file system and drop it on to the bottom or top editor

Does not work yet on iPad 4 and Android devices.
